# Rietveld refinement of AgCa_10_(PO_4_)_7_ from X-ray powder data

**DOI:** 10.1107/S1600536813007848

**Published:** 2013-04-05

**Authors:** Nataliya Yu. Strutynska, Igor V. Zatovsky, Ivan V. Ogorodnyk, Nikolay S. Slobodyanik

**Affiliations:** a Department of Inorganic Chemistry, Taras Shevchenko National University, 64/13 Volodymyrska str., 01601 Kyiv, Ukraine

## Abstract

Polycrystalline silver(I) deca­calcium heptakis(orthophos­phate), AgCa_10_(PO_4_)_7_, was obtained by solid-state reaction. It is isotopic with members of the series *M*Ca_10_(PO_4_)_7_ (*M* = Li, Na, K and Cs), and is closely related to the structure of β-Ca_3_(PO_4_)_2_. The crystal structure of the title compound is built up from a framework of [CaO_9_] and two [CaO_8_] polyhedra, one [CaO_6_] octa­hedron (site symmetry 3.) and three PO_4_ tetra­hedra (one with site symmetry 3.). The Ag^+^ cation is likewise located on a threefold rotation axis and resides in the cavities of the rigid [Ca_10_(PO_4_)_7_]^−^ framework. It is surrounded by three O atoms in an almost regular triangular environment.

## Related literature

For the structure of the mineral whitlockite, see: Calvo & Gopal (1975 ▶); Yashima *et al.* (2003 ▶). For powder diffraction studies and Rietveld refinements of phosphate-based whitlockite-related compounds, see: Lazoryak *et al.* (1996 ▶); Morozov *et al.* (2000 ▶, 2002 ▶); Zatovsky *et al.* (2007 ▶, 2010 ▶, 2011 ▶). For physical properties of these materials, see: Dou *et al.* (2011 ▶); Enhai *et al.* (2011 ▶); Lazoryak *et al.* (2004 ▶); Teterskii *et al.* (2005 ▶); Zhang *et al.* (2011 ▶). For the crystal structure of isotypic KCa_10_(PO_4_)_7_, see: Sandström & Boström (2006 ▶). For bond-valence calculations, see: Brown (2002 ▶).

## Experimental

### 

#### Crystal data

AgCa_10_(PO_4_)_7_
*M* *_r_* = 1173.46
Trigonal, 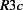

*a* = 10.43723 (5) Å
*c* = 37.3379 (7) Å
*V* = 3522.50 (7) Å^3^
*Z* = 6
Cu *K*α radiation, λ = 1.540560 Å
*T* = 293 K
Flat sheet, 25 × 25 mm

#### Data collection

Shimadzu LabX XRD-6000 diffractometer
Specimen mounting: glass container
Data collection mode: reflection
Scan method: step
2θ_min_ = 9.045°, 2θ_max_ = 100.045°, 2θ_step_ = 0.020°

#### Refinement

*R*_p_ = 0.094
*R*_wp_ = 0.125
*R*_exp_ = 0.042
*R*_Bragg_ = 0.051
*R*(*F*) = 0.038
χ^2^ = 8.821
4551 data points
150 parameters
3 restraints

### 

Data collection: *PCXRD* (Shimadzu, 2006 ▶); cell refinement: *DICVOL* (Boultif & Louër, 2004 ▶); data reduction: *FULLPROF* (Rodriguez-Carvajal, 2006 ▶); program(s) used to solve structure: *FULLPROF* (Rodriguez-Carvajal, 2006 ▶); program(s) used to refine structure: *FULLPROF* (Rodriguez-Carvajal, 2006 ▶); molecular graphics: *DIAMOND* (Brandenburg, 1999 ▶); software used to prepare material for publication: *PLATON* (Spek, 2009 ▶) and *enCIFer* (Allen *et al.*, 2004 ▶).

## Supplementary Material

Crystal structure: contains datablock(s) global, I. DOI: 10.1107/S1600536813007848/wm2726sup1.cif

Rietveld powder data: contains datablock(s) I. DOI: 10.1107/S1600536813007848/wm2726Isup2.rtv

Additional supplementary materials:  crystallographic information; 3D view; checkCIF report

## Figures and Tables

**Fig. 1.**
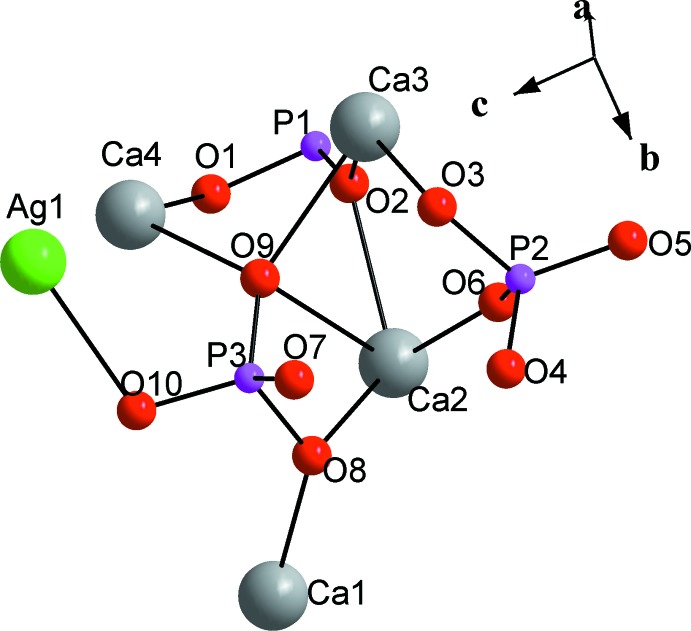
The asymmetric unit of the structure of compound (I).

**Fig. 2.**
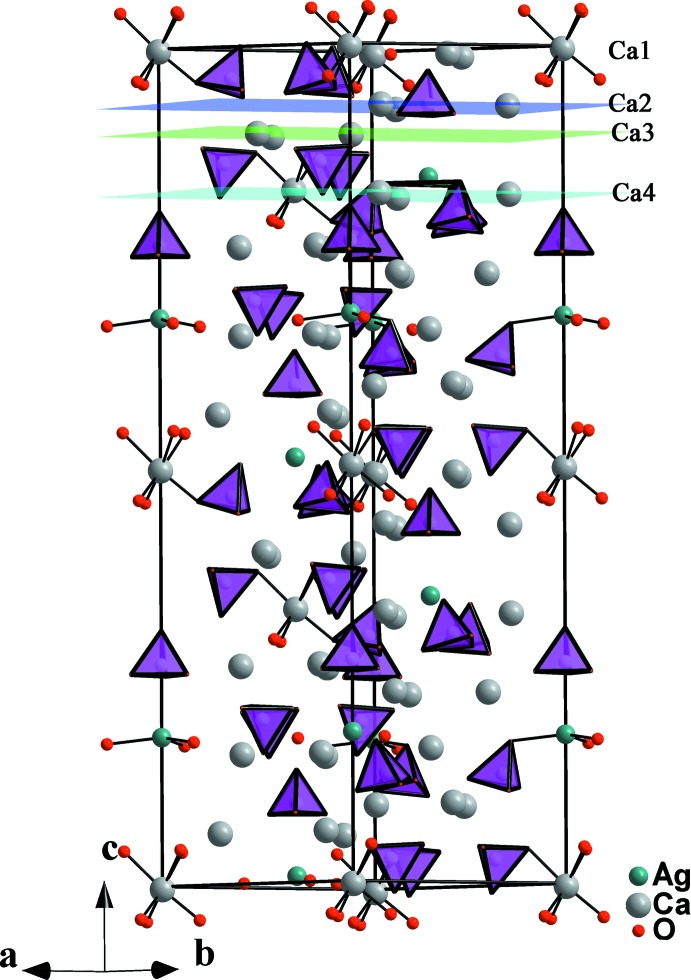
The environment of the four different Ca sites (violet plane corresponds to Ca2 sites, the green plane to Ca3 sites and the blue plane to Ca4 sites) and the Ag+ cation in the structure of (I). PO4 groups are represented as purple tetrahedra.

**Fig. 3.**
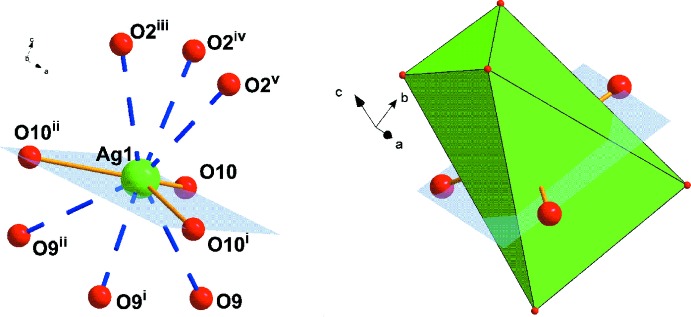
The coordination environment of the Ag+ cation. [Symmetry codes: (i) -x + y, -x, z; (ii) -y, x-y, z; (iii) -2/3 + x, -1/3 + x-y, 1/6 + z; (iv) 1/3 - y, 2/3 - x, 1/6 + z; (v) 1/3 - x + y, -1/3 + y, 1/6 + z].

**Fig. 4.**
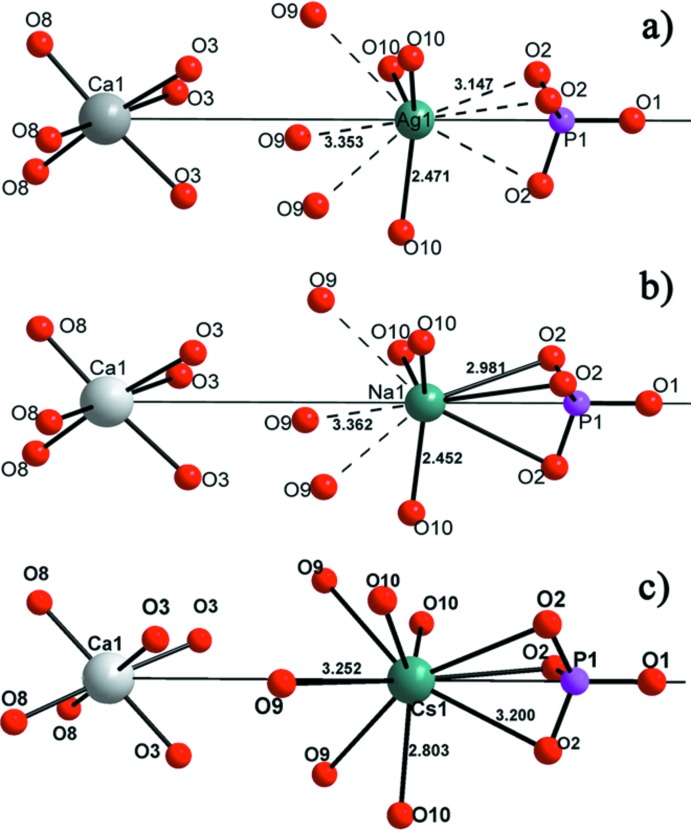
Comparison of the coordination environment of MI in the series MCa10(PO4)7; a) M = Ag; b) M = Na; c) M = Cs.

**Fig. 5.**
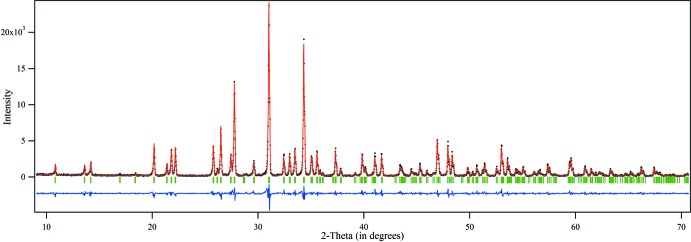
Rietveld refinement of AgCa10(PO4)7. Experimental (dots), calculated (red curve) and difference (blue curve) data for 2θ range 9–72°.

## supplementary crystallographic information

### Comment

In recent years phosphates which are isotypic with β-Ca_3_(PO_4_)_2_ (whitlockite; Calvo & Gopal, 1975; Yashima *et al.*, 2003) or whitlockite-related structures have attracted a growing interest due to their ferroelectric (Lazoryak *et al.*, 2004), non-linear optical (Teterskii *et al.*, 2005) or luminescent (Dou *et al.*, 2011; Enhai *et al.*, 2011; Zhang *et al.*, 2011) properties. The structure of β-Ca_3_(PO_4_)_2_ contains three phosphorus (P1—P3) and five metal (*M*1—*M*5) sites, that are amenable to different types of substitutions, thus yielding a large number of closely related compounds. The Ca sites in the *M*1 and *M*2 positions (6*a*) are prone to substitution by univalent metals under formation of *M*Ca_10_(PO_4_)_7_ compounds (*M* = Li, Na, K, Cs; Morozov *et al.*, 2000; Sandström & Boström, 2006; Zatovsky *et al.*, 2011), or by trivalent metals under formation of Ca_9_*M*(PO_4_)_7_ (*M* = Cr, Fe, In; Lazoryak *et al.*, 1996; Morozov *et al.*, 2002; Zatovsky *et al.*, 2007), or combinations of univalent and trivalent metals (Zatovsky *et al.*, 2010; Zatovsky *et al.*, 2011). The new title compound AgCa_10_(PO_4_)_7_, (I), is likewise isotopic to the family of *M*Ca_10_(PO_4_)_7_ (*M* = Li, Na, K, Cs) phosphates.

In the crystal structure of (I) four types of Ca sites (three in general positions 18*b* and one in special position 6*a*), three P sites (two in 18*b* one in 6*a*), ten O atoms (nine in 18*b* and one in 6*a*) and one Ag in 6*a* are present (Fig. 1).

The anionic framework [Ca_10_(PO_4_)_7_]^-^ of (I) is formed by interconnection of four types of [CaO*_x_*] and [PO_4_] tetrahedra (Fig. 2). The silver cations reside in cavities and compensate the charge of the rigid framework.

The Ca—O distances in the three types of [CaO*_x_*] polyhedra (one [CaO_9_] (Ca4) and two [CaO_8_] (Ca2, Ca3)) are in the range 2.28 (4)–2.97 (4) Å which is close to that in the series of *M*Ca_10_(PO_4_)_7_ structures (*M* = K, Cs; Sandström & Boström, 2006; Zatovsky *et al.*, 2011). The polyhedron [CaO_6_] (Ca1) is more irregular with Ca—O distances spread over the range 2.17 (4) to 2.40 (4) Å. In the case of *M*Ca_10_(PO_4_)_7_ (*M* = K, Cs), the corresponding distances are 2.23–2.31 Å. The nearest oxygen environment of the Ag site corresponds to an almost regular triangular arrangement. The position of the Ag site is slightly shifted by 0.30 (3) Å from the plane of the O_3_ triangle (Fig. 3). On both sides from the central triangular plane two further groups of Ag—O contacts can be observed. Three O2 atoms, which belong to a single orthophosphate tetrahedron, coordinate the Ag atom from one side of the plane and three O9 atoms, which belong to three different orthophosphate tetrahera, complete the other part of the [AgO_9_] coordination sphere. Such kind of arrangement of O atoms can be described as a distorted three-capped triangular antiprism (Fig. 3). The lengths of Ag—O contacts are 2.476 (19), 3.15 (4) and 3.35 (4) Å. In comparison with *M*Ca_10_(PO_4_)_7_ (*M* = Na, Cs) the corresponding *M*—O distances are: *d*(Na—O) = 2.452, 2.981, 3.362 (Morozov *et al.*, 2000) and *d*(Cs—O) = 2.803, 3.200, 3.252 Å (Zatovsky *et al.*, 2011) and the coordination numbers of the alkaline metal are six for Na and nine for Cs (Fig. 4(b,c)). For the Ag atom, the Ag—O2 distance (3.15 (4) Å) significantly exceeds that of Ag—O10 (2.471 (15) Å) thus indicating that the coordination number should rather be described as [3 + 6] (Fig. 4a). Bond valence calculations (Brown, 2002) of the Ag^+^ cation resulted in 0.60 valence units considering the three close O atoms, and 0.67 v.u. considering also the six remote O atoms, thus indicating a rather low contribution to the overall bonding of the latter O atoms.

### Experimental

The title compound has been prepared by solid state reactions from a mixture of Ag_3_PO_4_, CaCO_3_ and CaHPO_4_ in the stoichiometric molar ratio Ag:Ca:P = 1:10:7. The starting components were finely ground in an agate mortar and then placed in a porcelain crucible. The thermal treatment has been carried out in two steps. The first one included preheating to 873 K to decompose the carbonate and calcium hydrogen phosphate. After that, the mixture was annealed at 1173 K for 20 h. The final product was a white powder.

### Refinement

Structure refinement was performed using KCa_10_(PO_4_)_7_ (Sandström & Boström, 2006) as a starting model. For profile refinement Pearson VII function was used. For the oxygen atoms of each orthophosphate group the isotropic temperature factors were restrained as equal. The result of the final Rietveld refinement is given in Fig. 5.

### Crystal data

**Table:**

AgCa_10_(PO_4_)_7_	*D*_x_ = 3.319 Mg m^−^^3^
*M**_r_* = 1173.46	Cu *K*α radiation, λ = 1.540560 Å
Trigonal, *R*3*c*	*T* = 293 K
Hall symbol: R 3 -2"c	Particle morphology: isometric
*a* = 10.43723 (5) Å	white
*c* = 37.3379 (7) Å	flat sheet, 25 × 25 mm
*V* = 3522.50 (7) Å^3^	Specimen preparation: Prepared at 293 K and 101.3 kPa
*Z* = 6	

### Data collection

**Table:**

Shimadzu LabX XRD-6000 diffractometer	Data collection mode: reflection
Radiation source: X-ray tube, X-ray	Scan method: step
Graphite monochromator	2θ_min_ = 9.045°, 2θ_max_ = 100.045°, 2θ_step_ = 0.020°
Specimen mounting: glass container	

### Refinement

**Table:**

*R*_p_ = 0.094	150 parameters
*R*_wp_ = 0.125	3 restraints
*R*_exp_ = 0.042	3 constraints
*R*_Bragg_ = 0.051	Standard least squares refinement
*R*(*F*) = 0.038	(Δ/σ)_max_ = 0.001
χ^2^ = 8.821	Background function: Linear Interpolation between a set background points with refinable heights
4551 data points	Preferred orientation correction: March-Dollase Numeric Multiaxial Function
Profile function: Pearson VII	

### Special details

**Table:**

Geometry. Bond distances, angles *etc*. have been calculated using the rounded fractional coordinates. All su's are estimated from the variances of the (full) variance-covariance matrix. The cell e.s.d.'s are taken into account in the estimation of distances, angles and torsion angles

### Fractional atomic coordinates and isotropic or equivalent isotropic displacement parameters (Å^2^)

**Table:**

	*x*	*y*	*z*	*U*_iso_*/*U*_eq_	
Ag1	0.00000	0.00000	0.1780 (8)	0.042 (2)*	
Ca1	0.33333	0.66667	0.1632 (9)	0.002 (2)*	
Ca2	0.4650 (10)	0.5260 (11)	0.0955 (8)	0.0044 (14)*	
Ca3	0.2864 (7)	0.1558 (12)	0.0625 (8)	0.004 (2)*	
Ca4	0.3992 (5)	0.1876 (9)	0.1565 (8)	0.0044 (14)*	
P1	0.66667	0.33333	0.0976 (8)	0.002 (4)*	
P2	0.1577 (14)	0.3495 (13)	0.0288 (8)	0.009 (3)*	
P3	0.1366 (11)	0.3111 (7)	0.1306 (8)	0.003 (3)*	
O1	0.66667	0.33333	0.1387 (11)	0.006 (11)*	
O2	0.5229 (16)	0.325 (2)	0.0860 (9)	0.006 (6)*	
O3	0.082 (3)	0.1873 (15)	0.0421 (9)	0.005 (3)*	
O4	0.051 (2)	0.394 (3)	0.0420 (10)	0.005 (3)*	
O5	0.173 (2)	0.3689 (18)	−0.0111 (9)	0.005 (3)*	
O6	0.316 (2)	0.440 (2)	0.0462 (10)	0.005 (3)*	
O7	−0.0093 (19)	0.267 (3)	0.1100 (9)	0.006 (4)*	
O8	0.241 (3)	0.4824 (13)	0.1261 (9)	0.006 (4)*	
O9	0.221 (2)	0.243 (3)	0.1161 (10)	0.006 (4)*	
O10	0.0887 (18)	0.267 (2)	0.1700 (10)	0.006 (4)*	

### Atomic displacement parameters (Å^2^)

**Table:**

	*U*^11^	*U*^22^	*U*^33^	*U*^12^	*U*^13^	*U*^23^
?	?	?	?	?	?	?

### Geometric parameters (Å, º)

**Table:**

Ag1—O10	2.476 (19)	Ca3—O4^ii^	2.62 (4)
Ag1—O10^i^	2.476 (19)	Ca3—O6	2.89 (3)
Ag1—O10^ii^	2.476 (19)	Ca4—O7^ii^	2.40 (4)
Ca1—O8	2.17 (4)	Ca4—O6^ix^	2.45 (4)
Ca1—O8^iii^	2.17 (4)	Ca4—O4^vi^	2.46 (4)
Ca1—O8^iv^	2.17 (4)	Ca4—O1	2.510 (14)
Ca1—O3^v^	2.40 (4)	Ca4—O5^ix^	2.55 (3)
Ca1—O3^vi^	2.40 (4)	Ca4—O5^vi^	2.59 (2)
Ca1—O3^vii^	2.40 (4)	Ca4—O9	2.67 (4)
Ca2—O6	2.28 (4)	Ca4—O10^ii^	2.692 (18)
Ca2—O5^vi^	2.41 (4)	Ca4—O2	2.97 (4)
Ca2—O8	2.43 (4)	P1—O1	1.54 (5)
Ca2—O4^iii^	2.45 (4)	P1—O2	1.52 (2)
Ca2—O2	2.48 (3)	P1—O2^x^	1.52 (2)
Ca2—O8^iii^	2.48 (3)	P1—O2^xi^	1.52 (2)
Ca2—O7^iii^	2.57 (2)	P2—O3	1.55 (2)
Ca2—O9	2.88 (3)	P2—O4	1.49 (3)
Ca3—O7^ii^	2.31 (4)	P2—O5	1.50 (4)
Ca3—O3^ii^	2.37 (2)	P2—O6	1.58 (3)
Ca3—O2	2.37 (2)	P3—O7	1.56 (3)
Ca3—O9	2.43 (4)	P3—O8	1.570 (15)
Ca3—O3	2.44 (4)	P3—O9	1.48 (3)
Ca3—O10^viii^	2.46 (4)	P3—O10	1.55 (5)
			
O10—Ag1—O10^i^	118.5 (9)	O1—P1—O2^x^	106.6 (17)
O10—Ag1—O10^ii^	118.6 (7)	O2^x^—P1—O2^xi^	112.3 (18)
O10^i^—Ag1—O10^ii^	118.6 (8)	O2—P1—O2^x^	112.2 (16)
O7—P3—O8	107.7 (19)	O2—P1—O2^xi^	112.2 (17)
O7—P3—O9	114 (2)	O3—P2—O4	101 (2)
O7—P3—O10	105.0 (17)	O4—P2—O5	109 (2)
O8—P3—O9	105.5 (18)	O3—P2—O5	115.3 (19)
O8—P3—O10	112 (2)	O3—P2—O6	109 (2)
O9—P3—O10	112.7 (19)	O4—P2—O6	114 (2)
O1—P1—O2^xi^	106.5 (16)	O5—P2—O6	108.6 (19)
O1—P1—O2	106.5 (16)	Ag1—O10—P3	109.4 (15)

Symmetry codes: (i) −*y*, *x*−*y*, *z*; (ii) −*x*+*y*, −*x*, *z*; (iii) −*y*+1, *x*−*y*+1, *z*; (iv) −*x*+*y*, −*x*+1, *z*; (v) −*y*+1/3, −*x*+2/3, *z*+1/6; (vi) *x*+1/3, *x*−*y*+2/3, *z*+1/6; (vii) −*x*+*y*+1/3, *y*+2/3, *z*+1/6; (viii) −*y*+2/3, −*x*+1/3, *z*−1/6; (ix) −*x*+*y*+1/3, *y*−1/3, *z*+1/6; (x) −*y*+1, *x*−*y*, *z*; (xi) −*x*+*y*+1, −*x*+1, *z*.
